# Legal capacity, mental capacity and supported decision-making: Report from a panel event

**DOI:** 10.1016/j.ijlp.2018.09.006

**Published:** 2018-10-31

**Authors:** Jillian Craigie, Michael Bach, Sándor Gurbai, Arlene Kanter, Scott Y.H. Kim, Oliver Lewis, Graham Morgan

**Affiliations:** a Centre of Medical Law and Ethics, Dickson Poon School of Law, King’s College London, London WC2R 2LS, UK; b IRIS – Institute for Research and Development on Inclusion and Society, and Disability Studies, Ryerson University, Toronto, Canada; c Essex Autonomy Project, University of Essex, Colchester, UK; d Disability Law and Policy Program, Syracuse University College of Law, Syracuse, NY, USA; e Department of Bioethics, Clinical Center, National Institutes of Health, 10 Center Drive, 1C118, Bethesda, MD 20892-1156, USA; f Doughty Street Chambers, 53 Doughty Street, London WC1N 2LS, School of Law, University of Leeds, Leeds LS2 9JT, UK; g Mental Welfare Commission for Scotland, Thistle House, 91 Haymarket Terrace, Edinburgh EH12 5HE, UK.; h Institute for Disability and Social Participation, ELTE University, Budapest, Hungary

**Keywords:** Legal capacity, Mental capacity, Supported decision-making, Mental disability, UN Convention on the Rights of Persons with Disabilities, Article 12

## Abstract

Against a backdrop of the UN Convention on the Rights of Persons with Disabilities having been in place for over a decade, discussions about legal capacity, the relevance of mental capacity and the shift to supported decision-making, continue to develop. A panel event was held at the King’s Transnational Law Summit in 2018 with the aim of understanding the contours of the dialogue around these issues. This paper presents the contributions of the panel members, a summary of the discussion that took place and a synthesis of the views expressed. It suggests that divergent conclusions in this area turn on disagreements about: the consequences of sometimes limiting legal capacity for people with mental disabilities; the emphasis placed on particular values; the basis for mental capacity assessments; and the scope for supported decision-making. It also highlights the connection between resources, recognition and freedoms for people with mental disabilities, and therefore the issues that arise when discussion in this area is limited to legal capacity in the context of decision-making.

## 1. Introduction

The following account describes a panel event that was held as part of the King’s Transnational Law Summit at King’s College London in April 2018. Six commentators were invited to explore the use of the concept of ‘mental incapacity’ as grounds for restricting self-determination, against a background of the UN Convention on the Rights of Persons with Disabilities having been in place for over a decade (CRPD; UN General Assembly, 2006). This introduction outlines the context for the panel and the structure of this account.

By requiring that states recognise that “persons with disabilities enjoy legal capacity on an equal basis with others in all aspects of life”, the CRPD raises questions about law that can restrict personal decision-making powers due to a mental incapacity, for example, in decisions about medical treatment (2006, 12(2)). Exploring the obligations that derive from Article 12, General Comment No. 1 from the UN Committee on the Rights of Persons with Disabilities,^1^ held that the full and equal enjoyment of legal capacity for disabled people requires a shift to supported decision-making paradigms (2014, para. 3) and the abolition of substituted decision-making that allows forced treatment (2014, para. 7). In relation to the concept of mental capacity, the Committee held that “perceived or actual deficits in mental capacity must not be used as justification for denying legal capacity.” (2014 para. 13).

Uncertainty around the meaning of Article 12 is often said to have been settled by General Comment No. 1, for its interpretation is clear. However, the justifying reasons for this interpretation were not explored in depth. The Committee explains that its position is based on the guiding principles of the CRPD, which include: respect for inherent dignity, individual autonomy, independence of persons, non-discrimination, full and effective participation and inclusion in society, respect of difference and acceptance of persons with disabilities as part of human diversity and humanity (2014, para. 4). However, it is unclear how the step from these principles to the conclusions in General Comment No. 1, was made. Indeed, it seems plausible that the application of these principles might sometimes yield conflicting guidance that would need resolution. The requirements to respect the inherent dignity and value the independence of persons, for example, do not always point in the same direction. Much depends on the situation that is being considered, and how these principles are understood (Craigie, 2015).

The aim of the event was to understand the contours of the dialogue in response to the Committee’s interpretation of Article 12 of the CRPD. Sections 2 to 7 below summarise each panel member’s response to the questions: *What, if anything, justifies the position against mental incapacity as a basis for limiting legal capacity? Is there a place for mental capacity assessments within support-based legal capacity paradigms?* These sections begin with those exploring the reasons for the position in General Comment No. 1 and its implications, moving then to more critical perspectives on this position. Section 8 summarises the discussion that took place and reflects on a question about representation that arose at the event, before some concluding comments are drawn.

## 2. Arlene Kanter: Legal capacity and the CRPD

Are there any acceptable limits on equality and personhood? The CRPD responds to this question by stating that such limits may never be based on a person’s disability. Article 12 of the CRPD makes clear, for the first time under international law, that a person’s disability (or diagnosis) can not provide a justification for the denial of the person’s right to legal personhood or equal recognition under law.

Article 12 clarifies that persons with disabilities enjoy not only the equal right to legal capacity, as enjoyed by people without disabilities, but also the right to exercise their legal capacity on an equal basis with others. This article, therefore, calls for an end to substituted decision-making regimes that are included in most guardianship laws today. As the CRPD Committee has recognised: “Historically, persons with disabilities have been denied their right to legal capacity in many areas in a discriminatory manner under substitute decision-making regimes such as guardianship, conservatorship and mental health laws that permit forced treatment. These practices must be abolished in order to ensure that full legal capacity is restored to persons with disabilities on an equal basis with others.” (General Comment No. 1, para. 7).

For centuries, people with mental disabilities have been presumed to be incompetent, thereby justifying laws and policies that have deprived them of their rights to legal capacity and freedom – rights that people without disabilities take for granted. Today in most countries in the world, a person’s diagnosis provides the state with legal authority to appoint a guardian to make decisions for the person, without even consulting the person and without regard to the person’s preferences. A label of disability also provides the legal justification for involuntary confinement and treatment. The CRPD changes the legal presumption of incapacity that attaches to a label of disability and requires all people with disabilities to enjoy a presumption of competency and legal capacity, as a matter of human rights law.

Although guardianship and involuntary commitment laws were originally developed to protect persons who were considered unable to protect themselves (even without any evidence of past harm), these laws have neither ensured protection nor offered equal treatment for people with disabilities. People without disabilities are free to endanger themselves and others without state intervention – unless and until they violate criminal laws. To deny legal capacity and freedom to people on the basis of their disability, when they have broken no laws, is the very type of discrimination that the CRPD was drafted to address.

Both Articles 12 and 14 of the CRPD present an opportunity for State Parties to substitute the existing presumption of incompetency and incapacity for a presumption of competency and universal legal capacity. Yet the CRPD also recognises that with this new right of universal legal capacity must come the right to support (which I have argued elsewhere is now a new human right (Kanter, 2015, 2017)). Such support, as recognised in Article 12 (as well as in Article 19 on community living), will enable all persons to exercise their right to legal capacity, decision-making, and liberty. Without such supports, the right to equal recognition under law and universal legal capacity may not be realised.

The CRPD, therefore, invites us to envision the development of new legal protections to ensure that all persons enjoy the opportunity to exercise their right to decision-making and to communicate their decisions and preferences. If a person had been able in the past to communicate, then that past history is the guide for the person’s future decisions. If a person had never been able to communicate in the past but evidences some reasoning ability, then the law must provide an opportunity for that person to express his or her will and preferences. But if a person lacks reasoning ability and has never been able to communicate, then, and only then, must there be an opportunity for someone to make decisions affecting that person based on what would be that person’s will and preferences, as a practical matter, but without denying the person the right to legal capacity as a matter of law, and never only for the convenience of the supporter. Granted, such situations present new legal and moral challenges. But as the CRPD reminds us, such challenges must be met with responses that remain grounded, always, in the principles of equality and non-discrimination.

In response to this new model of universal legal capacity and support, several questions have arisen, only some of which I have the space to address here:

What if a person is chooses to endanger herself, can the state intervene to stop such danger? Does the state’s response differ if the person is about to physically endanger herself or others? Or if the harm is to property or financial loss to the person?

These are difficult questions that are not easily resolved, even with a careful reading of the CRPD and the CRPD Committee’s General Comments. Nonetheless, they call out for responses. One response is the development of disability-neutral laws to address situations of harm in place of current mental health and guardianship laws Such laws would apply to people with and without disabilities alike. As I have written elsewhere, even with disability-neutral laws, emergency situations may be identified in which legal agency can be overridden but in a way that does not use disability as the justification for intervention.

Moreover, even if we have not yet answered all of the questions left unresolved by the CRPD, it is time to consider new responses to some of society’s most challenging issues. Most countries throughout the world have relied on guardianship and mental health laws to respond to people who are considered at risk to themselves or others, or unable to care for themselves in a way that society accepts. Yet research has shown the fallacy of the premises of such laws as well as the problems with their implementation. We know, for example, that voluntary treatment is more successful than involuntary treatment, and that guardianship laws have resulted in many problems, and few, if any, solutions. For these reasons, alone, it is time to try a new regime of universal legal capacity and disability-neutral laws; indeed, that is what the CRPD demands.

## 3. Sándor Gurbai: An attempt to show that states must not impose restrictions on an individual’s legal capacity on the basis of mental incapacity

In this section I would like to address eight questions in order to be able to establish that states must not impose restrictions on an individual’s legal capacity on the basis of mental incapacity.

My first question is about *whether legal capacity of an adult person can be limited at all*. This question must be answered clearly in the affirmative since, for example, legal capacity can be limited as a consequence of imprisonment or bankruptcy. My second question is *whether legal capacity of an adult person can be limited on the basis of impairment or disability*. The use of the so-called status approach to limit legal capacity of a person with disabilities is increasingly rejected and does not appear as a question in many jurisdictions. The CRPD Committee also holds that a “person’s status as a person with a disability or the existence of an impairment (…) must never be grounds for denying legal capacity or any of the rights provided for in article 12” (2014, para. 9).

My third question can be identified as *whether mental incapacity can be a ground for imposing restrictions on legal capacity*. My answer to this question is ‘no’, mental incapacity cannot be accepted as a basis for imposing restrictions on legal capacity. If we turn to the CRPD Committee to identify *its* position on this question, we find that according to the treaty body of the CRPD, “perceived or actual deficits in mental capacity must not be used as justification for denying legal capacity” (2014, para. 13).

Now I have arrived at my fourth question: *What justifies the position against mental incapacity as a basis for imposing restrictions on legal capacity?* A number of different arguments have been advanced, but I propose here to rest my case on just one: the imposition of restrictions on legal capacity on the basis of mental incapacity constitutes discrimination. Let’s check what the opinion of the CRPD Committee about this is. In its General Comment on Article 12, the Committee points out that “The functional approach attempts to assess mental capacity and deny legal capacity accordingly. It is often based on whether a person can understand the nature and consequences of a decision and/or whether he or she can use or weigh the relevant information. This approach is (…) discriminatorily applied to people with disabilities (…)” (2014, para. 15).

At this point a fifth question must be answered too. *What is exactly claimed to be discriminatory: (i) Imposing restrictions on legal capacity on the basis of mental incapacity? Or (ii) Using mental capacity assessments in order to be able to decide whether to impose restrictions on legal capacity?*

The CRPD Committee emphasises that States Parties to the CRPD must abolish denials of legal capacity that are discriminatory on the basis of disability or decision-making skills (i.e. mental capacity) either in purpose (direct discrimination) or effect (indirect discrimination) (2014, para. 25). So, *what is discriminatory here is imposing restrictions on legal capacity on the basis of mental capacity and not mental capacity assessments*. However, something interesting is hidden in this paragraph of General Comment No. 1: *mental (in)capacity appears as a protected ground against discrimination*. A similar position is advanced in paragraph 15: “a person’s disability and/or decision-making skills are taken as legitimate grounds for denying his or her legal capacity and lowering his or her status as a person before the law. Article 12 does not permit such discriminatory denial of legal capacity.” (2014).

I cannot refrain from asking two more questions here: (1) *What is the reason why the CRPD Committee introduced the new protected ground of mental (in)capacity?* (2) *Can it be acceptable, and if yes why, that the CRPD Committee introduced a new ground, which is not included in the CRPD?* So my sixth question on my list is this: *What is the reason why the CRPD Committee introduced the new protected ground of mental (in)capacity?*

If the protected ground was only impairment or disability – without explicitly mentioning decision-making skills – then imposition of restrictions on legal capacity on the basis of mental incapacity could be identified as disability-neutral, and consequently only indirect discrimination could be claimed. However, when it comes to indirect discrimination, the State enjoys the so called objective justification defence and may show that the difference in treatment has a legitimate aim and the different treatment is necessary and proportionate in order to achieve the legitimate aim. Now, if ‘decision-making skills’ is a protected ground, then imposition of restrictions on legal capacity on the basis mental incapacity will be direct discrimination and, in my opinion, State Parties’ opportunity to justify different treatment is narrower in direct discrimination cases than in indirect discrimination cases when it comes to the framework established by the CRPD Committee (UN Committee on the Rights of Persons with Disabilities, 2018, para. 18(a)-(b)).

My joint seventh and eighth questions are these: *Can it be acceptable, and if yes why, that the CRPD Committee introduced a new ground, which is not in the CRPD?* I can see only one reason to answer ‘yes’ to this question (and here I am addressing option (ii) of my fifth question). Using mental capacity assessments in order to be able to decide whether to impose restrictions on legal capacity is discriminatory. The reason behind this answer is that mental capacity assessments are based on the presumption that adults with disabilities can be protected by imposing restrictions on their legal capacity. If restriction of legal capacity on the basis of mental capacity assessments was a widely accepted way of protecting adults, then it should be applied to adults with disabilities and adults without disabilities equally. However, this is not the case and the reasons behind this unequal treatment are deeply rooted preconceptions about and longstanding prejudices against persons with disabilities and especially persons with psychosocial and intellectual disabilities. I cannot agree with this approach.

I have to admit that a full critical assessment of my legal argument would require a more extensive examination of objections and a closer analysis of my claims. However, the limitations of space preclude a more detailed discussion of these here.

## 4. Michael Bach: When do people “require” support in exercising their legal capacity?

Article 12(3) of the CRPD recognises state obligations to ensure “access by persons with disabilities to the support they may require in exercising their legal capacity” (2006). But what does “require” mean in this context? Who determines what supports may be *required* in a specific decision-making context and on what basis? I want to address this question from the starting point of people with profound intellectual or cognitive disabilities. This is a group who would be found as mentally incapable under the cognitive tests of legal capacity extant in most jurisdictions. It is also a growing group of the population.^2^

Under Article 12 of the CRPD, and considering the General Comment No. 1 which followed (2014, para. 15), cognitive tests of legal capacity discriminate based on mental disability. However, what should happen if because of their cognitive disability a person is not able to make decisions for themselves, like provide informed consent for a health procedure, and no amount of support or accommodation will ever change that fact? Responses to this question can take a few forms:

- ‘These individuals require 100% support to exercise their legal capacity, and Article 12.3 obliges states parties to ensure access to supports that a person may require for this purpose.’ It’s just unclear what 100% support means.
- ‘We should let people act on their will and preference even if it puts them in a situation of serious harm, if it does not constitute criminal or civil negligence.’ But on what basis do we determine that a person ‘requires’ support where letting them act on an expression of their will and preferences, or letting them fail to act, because they cannot, could constitute civil negligence?
- ‘If we just gave these individuals enough time and support they would make their will and preferences known sufficient to make a specific legal decision.’ I think that position fails on a reality test when it comes to people with profound intellectual disabilities.
- ‘These are the difficult cases, and we should not be designing for a legal regime based upon them – remember, hard cases make bad law.’ However, on what basis are those ‘hard cases’ ruled outside the ambit of supports for legal capacity other than some version of a cognitive capacity standard?

What, then, are the building blocks of a more inclusive account of legal capacity? I have suggested elsewhere that a ‘capabilities approach’ to legal capacity can help address the limitations of the standard account of autonomy on which recognition of legal capacity is usually thought to rest (Bach, 2018; Bach & Kerzner, 2010). Tom Beauchamp and James Childress’ often-quoted version defines autonomy as “self-rule that is free from both controlling interference by others and from limitations, such as inadequate understanding, that prevent meaningful choice” (2009, 99) (Beauchamp and Childress, 2009). Autonomous action, they argue, takes place when a person acts: “(1) intentionally, (2) with understanding, and (3) without controlling influences that determine their action” (2009, 101). This standard cognitive account of autonomy is found in one form or another in various statements and guidelines on respecting autonomy and legal capacity.^3^

Consistent with an understanding that the ground of legal power is “manifest intention” (Lindahl & Reidhav, 2017), an inclusive approach to conceptualising decision-making capability would ground recognition of a person’s autonomy in the first dimension of the standard account – acting intentionally. Informed by the capabilities approach as Amartya Sen (1984) first conceptualised it within an equality rights framework, it would not require that individuals carry out steps 2) and 3) by themselves, as in the standard account. Consistent with a ‘planning theory of agency’ (Bratman, 1987, 2007; Shapiro, 2011), it would recognise that plans and decisions can be made with the input and support of others, who can interpret a person’s manifest intentions as the basis for making and executing needed decisions. Implementation would require safeguards to ensure that these plans and decisions are guided by valid and ‘best interpretations’ of decision-making supporters in the circumstances.^4^ In this way, decisions executed under such arrangements could meet the test of voluntariness.

This formulation suggests that the exercise of legal capacity still requires decision-making capability to be present – i.e. some understanding and appreciation of the nature and consequences of a decision – for example, for a health care decision. But the understanding and appreciation of a person’s manifest intentions and what they require in any decision-making context need not depend on a person having those abilities, if they reside in the minds of the decision-making supporters duly recognised to provide interpretive and other supports in the circumstances.

The formulation could be summarised as follows:

A person (P) is able to exercise legal capacity in a particular matter (M) when:

- P has directly manifest, or can reasonably be ascribed through interpretive support, a true intention that can serve as the basis for the reasoning and planning necessary to execute that intention in M;
- Where required, P has access to forms of decision-making support sufficient to translate that intention into an executable plan of action regarding M; and,
- Where needed, P has access to those forms of reasonable accommodations that are required to execute the plan of action that has its basis in P’s manifest intention.^5^

We should not shy away from recognising that some persons will, in fact, “require” supports to exercise legal capacity. In making these determinations, a capabilities approach to decision making, complemented with a ‘planning approach to agency’ provides an inclusive alternative to the cognitive test of capacity. It does so without giving up a coherent, albeit re-worked, account of autonomy on which the exercise of legal capacity can still firmly rest.

## 5. Graham Morgan: Compulsory treatment and capacity – Is anything more important?

A few weeks ago my compulsory community treatment order was renewed for the ninth year in a row, I have a diagnosis of paranoid schizophrenia; it means that I believe that I am a devil who is bringing about the end of the world. I have the most wonderful life with my partner; but, given the choice, I would stop taking my medication because I think it stops me realising just how evil I am. I feel that I need to face what I am and rid the world of someone like me. My friends, family and the professionals around me believe that without medication I will become very ill very quickly and very likely attempt to kill myself. Intellectually I can understand that I have an illness, can understand that it is schizophrenia. My mind understands that it is probably the medication and the support that helps me have the wonderful life I do, but my heart does not accept this. It says you have all made a mistake and that my every action is polluting the world and that I should be stopped destroying the people and places that I love.

I even sort of understand that I may lack medical capacity. I understand the basis for this assessment, and am in some ways grateful for the fact that I do not have to take the decision to stop my medication and lose the vibrant life that I have now.

I am able to exercise my legal capacity in almost all aspects of my life: while under compulsory treatment in the community, I have bought a house, got divorced, I have entered into a contract to have a book about my life published. And yet in one aspect of my life, my judgment is seen as profoundly impaired and in that aspect the state has removed my legal capacity. Is that right and proper? I think so; when I see friends who are in similar situations, I can see the need to intervene.

Let us consider my detention: I have an advance statement that describes how I would like to be treated if ever life falls apart again. I have chosen a named person to represent my best interests. I can appeal my compulsory treatment and if I wish, can have an advocacy worker help me represent my views and a lawyer represent me legally, I have the right to minimum intervention and I have the right to participate in my care in a way that is meaningful to me.

I have started off with a very controversial topic: for me, compulsory treatment in the community probably keeps me alive and yet I am aware there is limited evidence that this form of intervention is effective. However, I think there is ample evidence that detention in hospital when desperate, when you cannot bear another day, when the television is beaming horrific messages and instructions into your brain, is needed. Certainly there was when I was last in hospital, I remember the humiliation of running from the hospital with the alarms blaring, the nurses chasing me, cars slewing to a halt in front of me and the awfulness of being frogmarched back to the ward with six nurses flanking me, and yet my intention was to go to the beach, cover myself with petrol and set myself alight as I thought I would then become a loving spirit in the sky, helping the people I love. If left to exercise my legal capacity, I would have died and no peer support, no compassionate friend or loving professional, no aspect of Open Dialogue or eCPR would have made any difference.^6^ My death was my obsession over those months and yet now, after being helped, I am alive and at home and happy.

In 2016/17 the Mental Welfare Commission for Scotland spoke to over 250 people with mental health problems. A large majority of those people agreed that sometimes detention is necessary (Mental Welfare Commission for Scotland, 2017). The collective advocacy group for users of mental health services in the Highlands of Scotland, HUG (action for mental health), produced a report in 2015, that showed that 99% of the members they spoke with thought that we could lose the capacity to make decisions when ill and 87% thought we needed compulsorily treated in some circumstances (HUG, 2015).

In fact far from being radical many of their members say that, when suicidal, their judgment is almost always impaired, that when we are desperate to harm ourselves even when we know what we are doing, have, if you like, a rational reason for it, people still need to act to preserve life.

I am much more interested in improving supported decision making, where so much more could be done, all those areas where we could increase our autonomy and control and dignity and avoid the need for later detention.

I am struck by how friends, who have been detained frequently, have talked about how building up a trusting relationship with their helpers can stop not only compulsory treatment but later hospital admissions. When you know and trust someone and they do all they can to respect your life, which may be spotting the warning signs or making sure your children are looked after when you are manic, or knowing that you dissociate when in hospital and if, in the company of male nurses, are apt to be violent and that recognition of this can stop this from escalating, can stop the need for force.

To me, it is the ability of the system to respond to what we need, it is whether we can make hospitals and the community warm and caring places, detention something that is done with love that matters most.

In the Mental Welfare Commission for Scotland we are not asking the question of ‘Would you physically prevent your friend from leaping over the bridge when they are despairing and lost?’ because we think any right minded person would do that; instead we are asking what can be done within that despair to give as much choice and autonomy as possible. Personally I worry that this polarised debate detracts from a host of other issues, such as not being able to get support when suicidal because you are seen to have capacity and are expected to take responsibility for yourself at such times. People may suffer hugely from detention, but many people lose their lives, jobs and housing or are imprisoned for crimes because they cannot get the help they need and deserve at the point that they need it, whether they have capacity or not, are compulsorily treated or not.

## 6. Scott Kim: Deciding for others, with respect and care^7^

In answering the two panel questions, it may be helpful for the reader to know the perspective from which I am answering them.

A few years ago, I wrote a book on decisional capacity (Kim, 2010). An important aim of the book was to educate the reader on a progressive ‘modern’ functional conception of capacity, in contrast to the older diagnosis based model; indeed, I think that remains an important task. Looking back, however, there are parts of the book that I would have been able to express better had I been more familiar with the social model of disability; some parts now also sound too medicalised.

All of this is to say that, although I am coming from a medical perspective, I see that the medical ‘establishment’ can do a better job of incorporating valuable insights from the disabilities literature. I appreciate that the CRPD reflects a significant shift in the understanding of disability as sociopolitical, rather than merely medical, phenomena, and its importance for advancing and implementing disability rights worldwide (Sabatello & Schulze, 2014). Discussions and disagreements about Article 12 of the CRPD regarding mental and legal capacity must be considered in light of this (Appelbaum, 2016; Freeman et al., 2015; Scholten & Gather, 2018).

One domain in which a revision of the medical model of disability is clearly necessary is regarding persons who experience a condition such as Alzheimer’s disease. It is a highly stigmatised condition and there is no doubt that in its later stages creates immense challenges (thus, there is a limit to the social model also). But we as a society fail persons with Alzheimer’s disease when we do not provide sufficient resources—material, interpersonal, and cultural—to such persons and their families, perhaps because we are too focused on the medical aspects of this condition.

### 6.1. What, if anything, justifies the position against mental incapacity as a basis for limiting legal capacity?

It is true that mental capacity is a potentially dangerous basis for limiting someone’s decisional authority. This is because its assessment relies on broad criteria that are often unsettled in meaning, and requires clinical and (in the cases that go to court) judicial judgment. It can be a difficult task. Errors can be made. Without sufficient safeguards, abuse can occur.

I also agree with the underlying moral insight that may be driving the view that mental capacity should not affect legal capacity, namely, the insight that all human beings, regardless of their abilities and other characteristics, deserve full and equal respect as persons. I am in 100% agreement with the insight. I find philosophical views that tie ‘moral status’ to specific abilities extremely problematic. However, full and equal respect for persons seems to me a much more robust and demanding concept than legal capacity. It seems odd to insist that mental capacity (or any other ability or characteristic of a person) is unnecessary for full respect but then to insist that full respect requires actual exercise of ‘legal capacity.’

The best reason (and the only reason) why we sometimes need to make decisions *for* others—why we cannot jettison the concept of mental capacity altogether—is that it is just a basic fact that some people cannot make decisions for themselves in any commonly accepted sense of the word ‘decision.’ For example, it seems incontrovertible that a person lacks the capacity to make a treatment decision if her delirium leads her to believes that the doctors are in fact not doctors but impostors, and for this reason does not believe that what is being offered is a truly life-saving treatment.

### 6.2. Is there a place for mental capacity assessments within support-based legal capacity paradigms?

There is a place for mental capacity assessments in determining decisional authority because *there is no choice in the matter*. We can use words to describe things in a way that makes it seem there is an option of abolishing substituted decisions altogether. But if a person cannot make a decision, then even ‘allowing’ the person to make ‘her own decision with support’ is *in itself a decision made for that person* by someone else. Whether one appeals to a best interpretation of “will and preference” or of “best interests,” it is a basic fact that either way someone else and not the principal is proposing, adopting, and using the rule of interpretation. The same applies to whether we use substituted or supported decision-making. These rules are made and used by persons (an institutions and committees made up of persons) who are decisionally capable *for* others who are not, *regardless of which rule is used*.

But respect for persons requires that mental capacity assessments should be as infrequent as possible and that all mental capacity assessments must have, to the extent possible, a supported decision-making component. Returning to my example of persons with dementia, a common question raised in acute care hospitals is: Is this person safe to return home and is she competent to decide? But we should first ask: Are we supplying this person sufficient resources and support so that she can live as she wants, despite her limitations, in her familiar environment? In an ideal world, there would be sufficient support services and options available for disabled persons, and the need to assess a person’s capacity may rarely need to arise.

The debate should not be over whether we sometimes should decide for others. The fact is that we inevitably do, whether we acknowledge this or not. Unfortunately, whether we make decisions for others with the respect and care they deserve is not inevitable. That is the true, and very demanding, challenge we should be debating.

## 7. Oliver Lewis: Why isn’t the Committee’s interpretation of Article 12 universally accepted?

People with intellectual disabilities or psychosocial disabilities (mental health issues) have been stripped of their autonomy, segregated in far-away institutions, where their lives were cut short often by terrible conditions. They had no right to refuse psychiatric and other unwanted interventions, they had no equitable access to healthcare that they wanted and needed. If no family member was able to provide care, there was no access to independent living or the skills needed to give effect to it. People were stripped of the right to love, their children were removed arbitrarily. They were not allowed to vote or participate in public life. If they had any complaints about the above, they were denied access to justice. Lives were cut short. ‘Civil death’ describes how people were stripped not just of their autonomy, but of their rights.

That may be history in some countries, but in too many others that describes the current situation. It was against this reality that Article 12 of the CRPD was proposed as a response. Interestingly, the right to legal capacity was originally located in the same article as access to justice – two keys to unlock the door?

Article 12 calls for an end to guardianship regimes. It requires governments to radically rethink their approach. As a result of this global nudge, law and policy reform is underway in several countries, projects conceived of and driven by civil society organisations, many of whom were involved in developing the CRPD.

However, the interpretation by the CRPD Committee in General Comment No. 1 (GC1) goes further than recommending governments end guardianship. It calls on countries to abolish mental health laws and reduce coercion down to zero. In seeking to put an end to the horrors of guardianship, the interpretation of Article 12 has created a new problem: If states enacted laws that complied with GC1, harm would be usefully reduced in one group but shifted onto another group because the disabled person’s contemporaneous wishes and feelings would be determinative of any decision about them: finances, care, health, residence and contact with others.

The approach in GC1 makes sense within a model whereby a disability (whether that be a mental health issue, an intellectual disability, a condition of the brain such as dementia or brain injury) can never cloud a person’s decision-making. The Comment recommends that countries adopt laws that abandon the understand and appreciate test (set out, for example, in section 3(1) of the Mental Capacity Act 2005 in England and Wales). Instead, the Committee says, a person’s express or implied wishes and feelings are to be determinative, without asking further questions as to how the person formed their view or what factors they weighed up. This narrow type of autonomy is said to be more important than every other value: health, wellbeing, happiness, living a pain-free life and the right to non-discrimination. In some cases it may be, but in every scenario?

In 2006 when the convention was being negotiated, the provision that became Article 12 was hotly negotiated because it was so controversial. A footnote suddenly appeared that sought to undercut the provision’s meaning. The week in New York in August 2006 was tense and were it not for an agreement being reached with regard to Article 12, we would not have had a convention. When they got back home, the NGOs lobbied for the footnote to be deleted, and it was – Article 12 is part of an international treaty and should be treated as such. However, it should also be read in the context of how it was developed, which is in a fraught environment and the result of a political compromise.

A further thought on how the interpretation in CG1 was developed. The ethos of the participatory nature of the way the CRPD was developed has filtered through to the way that the Committee functions. This is to be welcomed. Since its establishment, disability activists have constituted the majority of Committee members. My hypothesis is that the closest ally for Committee members has been disabled persons’ organisations. That is to say, the stakeholder to whom the Committee pivots for advice is the NGO sector. This is different from other treaty bodies for whom governments are the grouping they are focused on, drawing from best practice examples and encouraging governments to implement laws that have been tried and tested elsewhere. I say all of this tentatively, because this is an impressionistic view that has not yet been researched.

There has been a furore among mental health professionals in particular about the way in which the committee failed to take into account of, or respond to, the many comments that were sent in after the draft was published and before the final version was published in 2014.^8^ People and organisations asking the Committee to acknowledge the complexity of legal capacity on the ground and to insert some nuance into its recommendations were ignored. As a result, their questions have gone unanswered and there has not been sufficient engagement in those vantages. In a sense, there is a stating of positions but insufficient dialogue to figure out ways of overcoming what is now a substantial impasse between states parties, all major service provider NGOs, most monitoring bodies, some people with disabilities on one hand and the Committee supported by the main global disability rights NGOs on the other.

Although GC1 provides some valuable insights into autonomy and the role of supports, it is perhaps less helpful as a law reform tool. Article 12 has provided a clear direction of travel, a clear push of the swing from protection to autonomy. However, some worry that the solutions offered by the Committee are unfeasible. If that is right there is little hope of domestic law coming into ‘full compliance’ with the Committee’s interpretation of Article 12. This may result in people with disabilities having false hope. Structurally, an interpretation by a UN body that is not feasible risks undermining the authority of the wider UN human rights machinery. It is likely to be unprecedented in international human rights law for a treaty body to articulate as a norm, something that is not reflected in a law anywhere. It is not clear how to move beyond the impasse, apart from insisting on dialogue and having a diverse range of views around the table.

## 8. Jillian Craigie: Discussion and the challenge of representation

This final section provides an outline of the questions that were put to the panel and the discussion that followed.^9^ It also raises an issue that came to light in conversations directly after the event, which concerns the divergent perspectives on issues of legal capacity among those with experience of psychosocial disability. This issue is considered, briefly, in the wider context of debates about representativeness and the role of non-governmental organisations (NGOs) in international treaty making.

The audience members were not required to register for the panel event so we don’t have a picture of who was present. However, based on those who identified themselves in the discussion, questions came from service user, disability advocacy, clinical and academic perspectives.^10^

The discussion began with a question voicing concern that people with leaning or mental health difficulties, particularly women, face significant barriers in accessing justice when it comes to the context of domestic violence. This was acknowledged by Oliver Lewis as a serious problem, showing how the issue of legal standing also arises in criminal law, for example when cases aren’t prosecuted because a person is not considered a credible witness due to a mental disability.

This was followed by a question about whether the disagreements among the panel members about the concept of ‘mental capacity’ are less fundamental than it may seem, once we recognise that mental capacity is never wholly determinative in decisions about legal powers. An example was provided from criminal law, where the concept of intention plays a role in questions of responsibility, but does not settle the question because public policy considerations shape its role. For example, where public health and safety is at risk due to pollution or the sale of unsafe meat, a strict liability rule can apply, making an intention to commit the crime not necessary for a finding of responsibility.^11^ It was suggested that, in a parallel way, the crucial issue is what role mental capacity should play in relation to legal powers, and in order answer this we need to ask what this area of law trying to achieve.

A number of public policy issues were raised in response. Scott Kim suggested that it is a mistake to think that the highest value in this area should be assigned to giving decisional authority to the person, as implied by the reading of Article 12 in General Comment No 1. Michael Bach made the point that where there is agreement on an issue of mental capacity, there may nonetheless be disagreement about what follows. He would argue that although a person might not be able to make a decision by themselves—for example, because they lack the ability to understand—it does not follow that they cannot have any power over the decision. What’s required is law that recognises translations of intentions and mere manifestations of will, into decisions. However, he suggested, this won’t be achieved by automatically giving legal consequence to contemporaneous expressions of will. On these points, Graham Morgan read the words of another, who, in connection with experience of involuntary detention and treatment, reported relief at a diffusion of responsibility, a feeling of safety and a sense of these interventions as a means of receiving treatment they would otherwise be unable to accept.

The next question raised the criticism that in many of the contexts being discussed, issues of care seem at least as relevant as issues of legal and mental capacity, and are being overlooked. Scott Kim agreed that debates about legal capacity have diverted intellectual and policy energies from, in his view, more important issues around the provision of care. Graham Morgan made the point that in discussions with members of HUG, a collective advocacy group for users of mental health services in the Highlands of Scotland, people’s priorities concerned the preservation of existing services; the improvement of services and access to help; addressing stigma and discrimination; and addressing financial problems. These issues, he observed, affect freedom in a different way. Panel moderator, Alex Ruck Keene, noted the individualistic values reflected in the narrow focus on consent in discussions around the CRPD, and the importance of also focussing on the provision of services and resources, to minimise the circumstances when questions of mental and legal capacity arise.

One final question concerned whether a shift from a focus on the nature and degree of a diagnosis as justification for involuntary treatment, to a focus on mental capacity, would improve the situation of disempowerment for someone who wants to refuse treatment. A similar concern was expressed by another audience member after the panel, who suggested that mental capacity assessments are significantly based on diagnostic status. Scott Kim’s response was that a mental capacity approach enables others to recognise the potential reasonableness of a refusal, which is important to take seriously. For example, drugs can have significant side-effects. However, he pointed out, working with someone to do things differently, takes time and resources and trusting relationships. Once again, the link between access to care and resources, and empowerment and recognition, was brought to the fore.

Following the discussion, a few audience members expressed concerns to the moderator and I that their perspectives as survivors of psychiatry had not been represented on the panel. The issue of representation seemed important to raise here because those concerns were strongly felt, but also because I believe that this is a central challenge for this area, which needs to be more broadly acknowledged and explored. The audience members’ objection was not, as I understood it, that their views about mental and legal capacity were absent from the event, but rather that the service user voice on the panel did not represent their experience or interests.

Within the microcosm of this event, the lesson for me was about the importance of representing, from a first-person perspective, the diverse views among those who have been or may be subject to the relevant laws.^12^ The wider issue concerns whether the full range of service user voices are being heard in national and international discussions in this area. Reports of the CRPD negotiations around legal capacity speak of the “vigorous advocacy against compulsory treatment” by service user activists (Kayess & French, 2008, p.29), and the influential role played by the World Network of Users and Survivors of Psychiatry (WNUSP) (Minkowitz, 2015). The participation of such organisations in the CRPD negotiations has been described as “a significant breakthrough in efforts to democratize global health governance” (Lord, Suozzi, & Taylor, 2010, p.564, 567), “in a way that has not occurred previously on the international stage” (Kanter, 2015, p. 302). However, the increasing prominence of NGOs in international treatymaking has also raised questions about the basis of their authority and related issues about representativeness (Raustiala, 2012; Webb, forthcoming). Responding to the position on legal capacity advocated by WNUSP in the drafting and interpretation of the CRPD, Anne Plumb asks, “How have sections of the service-user/survivor constituency, people like myself and participants in Katsakou’s research, been so side-lined?” (Plumb, 2015, p.195, Katsakou et al., 2012).

As states and organisations grapple with implementing the CRPD in areas concerning legal capacity, there needs to be wider acknowledgment of the diversity of views among people with intellectual or psychosocial difficulties, on these issues. Anne Plumb has called for “more collective dialogues” among service-users/survivors to develop “clear demands” that leave no-one feeling overlooked (2015, p.198). However, given that disagreement among service users on issues of legal capacity seems likely to persist, thought must also be put into the challenge of how these differences can be reflected and reconciled in the development of law, policy and practice.

## 9. Concluding comments

The perspectives outlined in this paper do not seek to be representative of the many stakeholders who have expressed views regarding the interpretation of Article 12 of the CRPD and how legal capacity can be operationalised in various contexts around the globe. However, these perspectives do illustrate a range of views, and this conclusion identifies the key ideas that were expressed. Each of these ideas deserves deeper exploration than was possible in this paper. Nonetheless, the hope is that presenting them here in this way, brings into focus some of the issues that are at stake and must be given consideration, in the round.^13^

Concerns about mental incapacity-based infringements on legal capacity centred around discrimination against people with mental disabilities, which is said to diminish a person’s status before the law; the idea that mental incapacity regimes are rooted in misguided preconceptions about people with psychosocial or intellectual disabilities, which presume the person is unable to make decisions; that mental capacity assessments are based on criteria with unsettled meaning, requiring clinical or judicial judgment where mistakes can be made; or, more strongly, that mental capacity assessments are significantly based on diagnostic status; a claim that such regimes have not ensured protection for people with mental disabilities; and that they do not adequately recognise that some abilities necessary for making a decision need not rest in the cognitive abilities of the person, but can be provided though decision-making support.

Reasons given for the legitimacy of mental capacity-based approaches, in some form, included the necessity to be just as committed to respecting and protecting the inherent right to life, and stepping in to save someone’s life when they are severely distressed, as protecting their right to legal capacity; the idea that this imperative to step in can take priority, in some very restricted circumstances, over the personal indignity and restricted self-determination suffered as a result; a concern that discussion in this area has adopted an individualistic orientation that obscures a wider set of important values and human rights; the view that mental capacity-based regimes allow others to recognise the reasonableness of a decision such as a treatment refusal; and a claim that sometimes no amount of support will enable a person to make a particular decision with mental capacity, so whether it is recognised or not, decisions will sometimes be made by others.

Finally, the connection between resources, recognition and freedoms for people with mental disabilities arose at several points during the event, and concern was expressed that a narrow focus on legal capacity in the context of decision-making is causing these other important issues to be overlooked. Issues of resources and care are crucial because readily available, high-quality services and support^14^ can minimise the circumstances when concerns about legal capacity arise, and enable the exploration of alternatives that value well-being and the recognition of legal personhood, when they do.

## Footnotes

1 Hereafter, the CRPD Committee or Committee.

2 For example, Alzheimer’s Disease International (2015) estimates that the number of people living with dementia will double every 20 years from 46.8 million 2015, reaching over 130 million in 2050. While not all people with dementia or intellectual disabilities have ‘profound’ cognitive disabilities, many in these groups do, and their legal capacity is often questioned.

3 For example, see Ministry of the Attorney General of Ontario, Capacity Assessment Office (2005).

4 The UN Committee on the Rights of Persons with Disabilities (2014, para. 21) recognises the “best interpretation of will and preferences” as a standard to guide decision-making supporters in assisting persons in these situations to exercise legal capacity.

5 I gratefully acknowledge Professor Wayne Martin, University of Essex, who provided helpful critical feedback and suggestions in arriving at this summary formulation of a capabilities approach to defining the conditions for recognising and enjoying legal capacity, as an alternative to the predominant account of ‘mental capacity’ as its necessary condition

6 ‘Open Dialogue’ is a mental health care model that involves a family and social network approach in which treatment is carried out via whole network meetings, which always include the patient. Emotional CPR (eCPR) is an educational program designed to teach people to assist others through an emotional crisis by three steps: C = Connecting P = empowering and R = Revitalising.

7 The ideas and opinions expressed are the author’s own; they do not represent any position or policy of the National Institutes of Health, the Department of Health and Human Services, or the U.S. government.

8 The array of critiques includes submissions by the Canadian Association for Community Living, the Federal Republic of Germany, Dr. Lucy Series, the Mental Disability Advocacy Centre and Dr. George Szmukler at https://www.ohchr.org/EN/HRBodies/CRPD/Pages/DGCArticles12And9.aspx

9 Professor Arlene Kanter was unable to attend the event in person, which is why she is not present in the discussion below.

10 The audience was made aware that the event was being audio recorded. However, because consent was not sought for the identification of audience members or their organisations in a publication, the identity of those who asked questions has not been given in any detail.

11 Other reasons that are given for adopting a strict liability approach, removing the mental element of intention from a crime, include the aim of increasing the conviction rate, providing a strong deterrence or making the relevant offences easier to administer.

12 See: Mental Health Alliance (2017), Katsakou et al. (2012), Sheehan and Burns (2011), Owen et al. (2009), Gardner et al. (1999), Lidz (1998). A helpful review of other research in this area, across a range of jurisdictions, is forthcoming in a chapter by Campbell, Davidson, & Morgan (forthcoming 2018). I provide these as examples of research investigating service user views in this area, while noting that concerns have been raised, for example, about the methods used in the Mental Health Alliance report (personal communication).

13 It was suggested by one of our reviewers that the perspectives expressed in these two sets of considerations indicate two different approaches to realising the equal and non-discriminatory enjoyment of human rights. Because this issue was not raised in the contributions or discussion it hasn’t been included here, but it is noted as something to be considered when reflecting on these views.

14 ‘Services and support’ is understood here in a wide way that would include areas such as housing, employment and relationships, which can play a crucial role in a person’s mental health, in addition to the provision of advocacy, crisis houses, psychological therapies and medical services.

## References

[R1] Alzheimer’s Disease International (2015). World Alzheimer Report 2015. The Global Impact of Dementia: An analysis of prevalence, incidence, cost and trends. https://www.alz.co.uk/research/world-report-2015, Accessed date: 20 June 2018.

[R2] Appelbaum PS (2016). Protecting the rights of persons with disabilities: An international convention and its problems. Psychiatric Services, 67(4), 366–368. 27032795 10.1176/appi.ps.201600050

[R3] Bach M (2018). Vulnerability to losing legal power over personal life: A disability rights framework for analysis. Toronto: IRIS Institute for Research and Development on Inclusion and Society (forthcoming).

[R4] Bach M, & Kerzner L (2010). A new paradigm for protecting autonomy and the right to self determination. Toronto: Law Commission of Ontario.

[R5] Beauchamp TL, & Childress JF (2009). Principles of biomedical ethics (6th ed.). New York: Oxford University Press.

[R6] Bratman M (1987). Intentions, plans and practical reason. Cambridge, Mass: Harvard University Press.

[R7] Bratman M (2007). Structures of agency. New York: Oxford University Press.

[R8] Campbell J, Davidson G, & Morgan G (2018). Safeguarding those experiencing mental ill-health. In Macintyre G, Stewart A, & McCusker P (Eds.). Safeguarding adults, key themes and issues London: Palgrave (forthcoming).

[R9] Craigie J (2015). Against a singular understanding of legal capacity: Criminal responsibility and the Convention on the Rights of Persons with Disabilities. International Journal of Law and Psychiatry, 40, 6–14. 25997381 10.1016/j.ijlp.2015.04.002 PMC4503818

[R10] Freeman MC, Kolappa K, de Almeida JM, Kleinman A, Makhashvili N, Phakathi S, … Thornicroft G (2015). Reversing hard won victories in the name of human rights: A critique of the General Comment on Article 12 of the UN Convention on the Rights of Persons with Disabilities. Lancet Psychiatry, 2(9), 844–850. 26236004 10.1016/S2215-0366(15)00218-7

[R11] Gardner W, Lidz C, Hoge S, Monahan J, Eisenberg M, Bennett N, … Roth L (1999). Patients’ revisions of their beliefs about the need for hospitalization. American Journal of Psychiatry, 156(9), 1385–1391. 10.1176/ajp.156.9.1385 10484949

[R12] HUG (2015). Detention under the mental health act. http://www.spiritadvocacy.org.uk/assets/Detention-under-the-Mental-Health-Act-2015.pdf, Accessed date: 12 June 2018.

[R13] Kanter AS (2015). The development of disability rights under international law: From charity to human rights. New York: Routledge.

[R14] Katsakou C, Rose D, Amos T, Bowers L, McCabe R, Oliver D, … Priebe S (2012). Psychiatric patients views on why their involuntary treatment was right or wrong a qualitative study. Social Psychiatry and Psychiatric Epidemiology, 47, 1169–1179. 21863281 10.1007/s00127-011-0427-z

[R15] Kayess R, & French P (2008). Out of darkness into light? Introducing the convention on the rights of persons with disabilities. Human Rights Law Review, 8(1), 1–34.

[R16] Kim SYH (2010). Evaluation of capacity to consent to treatment and research. New York: Oxford University Press.

[R17] Lidz CW (1998). Coercion in psychiatric care: What have we Learned from research? The Journal of the American Academy of Psychiatry and the Law, 26(4), 631–637. 9894219

[R18] Lindahl L, & Reidhav D (2017). Legal power: The basic definition. Ratio Juris, 30(2), 158–185.

[R19] Lord J, Suozzi D, & Taylor A (2010). Lessons from the experience of the U.N. Convention on the Rights of Persons with Disabilities: Addressing the Democratic Deficit in Global Health Governance. Journal of law, Medicine & Ethics, 38(3), 564–579. 10.1111/j.1748-720X.2010.00512.x 20880241

[R20] Mental Health Alliance (2017). A mental health act fit for tomorrow: an agenda for reform. http://www.mentalhealthalliance.org.uk/news/A_Mental_Health_Act_Fit_For_Tomorrow.pdf, Accessed date: 12 June 2018.

[R21] Mental Welfare Commission for Scotland (2017). Seeking your views consultation: capacity, detention supported decision making and mental ill health. https://www. mwcscot.org.uk/media/371015/capacity__detention__supported_decision_making_and_mental_ill_health.pdf, Accessed date: 12 June 2018.

[R22] Ministry of the Attorney General of Ontario, Capacity Assessment Office (2005). Guidelines for conducting assessments of capacity. http://www.attorneygeneral.jus.gov.on.ca/english/family/pgt/capacity/2005-05/guide-0505.pdf, Accessed date: 20 June 2018.

[R23] Minkowitz T (2015). Advancing the rights of users and survivors of psychiatry using the UN Convention on the Rights of Persons with Disabilities. In Spandler H, Anderson J, & Sapey B (Eds.). Madness, distress and the politics of disablement (pp. 171–182). Bristol: Policy Press.

[R24] Owen GS, David AS, Hayward P, Richardson G, Szmukler G, & Hotopf M (2009). Retrospective views of psychiatric in-patients regaining mental capacity. British Journal of Psychiatry, 195, 403–407. 10.1192/bjp.bp.109.065151 PMC2806572 19880929

[R25] Plumb A (2015). UN Convention on the Rights of Persons with Disabilities: Out of the frying pan into the fire? Survivors aligning with the disability movement. In Spandler H, Anderson J, & Sapey B (Eds.). Madness, distress and the politics of disablement (pp. 183–198). Bristol: Policy Press.

[R26] Raustiala K (2012). The role of NGOs in international Treaty-making. In Hollis D (Ed.). The Oxford guide to treaties (pp. 150–175). Oxford: Oxford University Press.

[R27] Sabatello M, & Schulze M (2014). Human rights and disability advocacy (1st ed.). Philadelphia: University of Pennsylvania Press.

[R28] Scholten M, & Gather J (2018). Adverse consequences of article 12 of the UN Convention on the Rights of Persons with Disabilities for persons with mental disabilities and an alternative way forward. Journal of Medical Ethics, 44(4), 226. 29070707 10.1136/medethics-2017-104414 PMC5869457

[R29] Sen A (1984). Resources, values and development. Cambridge, Mass: Harvard University Press.

[R30] Shapiro S (2011). Planning agency and the law. In Bertea S, & Pavlakos G (Eds.). New essays on the normativity of law (pp. 17–72). Oxford: Hart Publishing.

[R31] Sheehan KA, & Burns T (2011). Perceived coercion and the therapeutic relationship: A neglected association? Psychiatric Services, 62(5), 471–476. 21532071 10.1176/ps.62.5.pss6205_0471

[R32] UN Committee on the Rights of Persons with Disabilities (2014). General comment no. 1, article 12: Equal recognition before the law, CRPD/C/GC/1. http://daccessdds-ny.un.org/doc/UNDOC/GEN/G14/031/20/PDF/G1403120.pdf?OpenElement, Accessed date: 20 June 2018.

[R33] UN Committee on the Rights of Persons with Disabilities (2018). General Comment No. 6, Article 5: Equality and non-discrimination. http://tbinternet.ohchr.org/_layouts/treatybodyexternal/Download.aspx?symbolno=CRPD/C/GC/6&Lang=en, Accessed date: 20 June 2018.

[R34] UN General Assembly (2006). Convention on the rights of persons with disabilities, 13 December, A/RES/61/106. https://www.un.org/development/desa/disabilities/convention-on-the-rights-of-persons-with-disabilities/convention-on-the-rights-of-persons-with-disabilities-2.html, Accessed date: 20 June 2018.

[R35] Webb P (forthcoming). The participation of non-state actors in the multilateral treaty process. In Malone D, Chesterman S & Villalpando S (Eds.), Oxford Handbook of United Nations Treaties. Oxford: Oxford University Press.

